# Where is genomics going next?

**DOI:** 10.1186/s13059-019-1626-2

**Published:** 2019-01-22

**Authors:** Barbara Cheifet

**Affiliations:** BioMed Central, New York, NY USA

## Abstract

We polled the Editorial Board of *Genome Biology* to ask where they see genomics going in the next few years. Here are some of their responses.

## Main text

Many fascinating studies were published in 2018 in all areas of genomics. As datasets get larger, there are no limits to the number of biological insights that can be found, but it has also been clear that we need novel and improved methods to help us analyze these datasets. We anticipate that 2019, and the years that follow, will see huge strides in genomics. What should we look out for? *Genome Biology*’s editorial board has some ideas.

## Rob Knight, University of California San Diego

Genomics is a key underpinning for metagenomics. This is the case because reference-based approaches are dramatically faster and more accurate than reference-free approaches whenever the reference database is complete and correct. However, with a few exceptions (such as bacteria in the human gut of healthy Western adults), we are far from having adequate reference data. Strain-sequencing efforts such as the Genomic Encyclopedia of Bacteria and Archaea (GEBA) projects have been extremely valuable in filling in missing branches of the tree of life, but projects such as Microbial Earth, which seeks to sequence all type strains, and the 1000 Fungal Genomes project remain under-resourced. Building these references and augmenting them with new clinical isolates and with isolates from remote human populations and from a panel of diverse environmental samples, such as those provided by the Earth Microbiome Project, could dramatically accelerate progress in all metagenomic studies, whether targeted at human or animal health or at the environment. The benefits that could be achieved would greatly outweigh the modest investment required to complete these studies.

## Mihaela Zavolan, University of Basel

I think that the near future will bring an increasing amount of data (from individuals and different cell types, for example) and data of higher resolution (from single cells or subcellular localizations). There are many challenges that accompany increased resolution. For every new dimension that we add, the complexity of the datasets that we need to infer patterns is multiplied. If we want to relate different types of measurements, we need to carry out a lot of new experiments in these systems. Integrating the data also becomes very difficult. The intuitive way of doing that would be through mechanistic models, but we do not have good models with well-defined parameters at the scale of cells or organs. What we clearly need are automated methods to process large datasets rapidly and robustly, and then accurate models to identify drivers of gene expression patterns and to map the flow of information in time and across organs upon perturbation.

## Sang Yup Lee, Korea Advanced Institute of Science and Technology

The cost of sequencing a human genome has dropped from $2.7 billion in 2003 to approximately $1500 in 2018. In addition, an increasing number of people are using direct-to-consumer genetic tests that analyze partial yet interesting genome regions at a cost of less than $100. In the coming era of precision medicine, more and more people will be obtaining their own whole-genome sequence. Data security, data sharing, and data ownership issues will become essential factors to be considered in the light of universal human rights. Blockchain and related technologies might play increasingly important roles in addressing these factors. We are moving into the era of genomics-based healthcare. Further into the future, living styles and environmental conditions together with epigenomes and microbiomes will be further integrated into human genomic studies to help establish a better healthcare system.

## Hong Ma, Fudan University

I believe that, increasingly, genomics will be integrated with other biological studies, such as studies of evolution, comparative genomics, transcriptomics and other omics as well as large-scale functional studies.

I can suggest a few specifics:

1. Structural genomics will increasingly involve the comparison of multiple genomes rather than single-species genomics. These comparisons can be quite comprehensive, offering insights above those that can be achieved by studies of a number of genes alone, or by syntenic analysis, or through deduction of ancestral genomes.
2. Genome-wide analysis of evolutionary questions, such as studies of genes that are important for development or physiology or studies of the evolutionary genomics of human diseases that have a complex genetic basis, such as heart disease and diabetes.
3. Genome biology of cis regulatory elements and of non-coding RNAs, with reference to functional studies and comparative analyses.
4. Comprehensive analysis of the dynamic properties of genomes, of the spatial properties of genomes, of changes in single-cell genomes, and of the relationships between these features and their functions.

For these studies, we need more computational tools and we need more experimental biologists to contribute to genomics. Even further, we need a new generation of biologists who have training in both experimental approaches and computational expertise.

## Jernej Ule, The Francis Crick Institute

The genomics field has matured, and the emphasis could now be shifting from methods-focused approaches to more theory-focused approaches, with stronger roles for machine learning in uncovering underlying principles. High-throughput sequencing and methods that rely on it have been the main driving force of genomics in the past decade.

In the future, hypotheses that are based on genomic data could be investigated more thoroughly, for example with the use of CRISPR-Cas-based approaches. I expect that the integration of genomics data with orthogonal information will become crucial, especially with regard to data from imaging, proteomics, and biophysics with purified components. We will be able to move beyond a static picture of genomic data towards studies of the dynamic transitions that cells make on a genomic scale in response to external and internal cues.

Manuscripts that present a single primary finding or method tend to have an easier time with editors and reviewers, as a single, linear storyline is easier to write and present. However, we need more multidimensional, theory-based, integrative work. Support from the editors of such work will be essential to drive the field further.

## Olivier Harismendy, University of California San Diego

### Implementation of genomic medicine

There is still a large disconnect between the way we use genomic information for biomedical research and the way we use it in healthcare. A lack of clinical utility and hence a rationale for insurance coverage is often brought forward, but I foresee a slow change in this landscape as large employer-payers (in the US) start to understand the benefits of genomic medicine for their employees and begin to contribute to accumulating evidence for this. From a technical standpoint, a large effort to standardize clinical genetics reporting is still required. At present, multiple initiatives are trying to find common ground to allow systems that are operated by providers and hospitals to be interoperable. Such standardization will make the reporting and use of genomic information really easy and friendly for doctors and patients, and will also provide opportunity for large data-sharing initiatives to continue the accumulation of evidence. Such development will be primarily supported by large initiatives such as the UK-biobank and the All of Us program.

### Sequencing technologies

There are still many weaknesses in the way that we generate and interpret sequencing data today. Short-read technology is ubiquitous but still has issues with interrogating certain portions of the genome or transcriptome, sometimes leading to false-positive results or spurious functional associations. The reliance on a universal human genome reference is also a weakness that may impair our ability to study human genetic diversity extensively. Large, ethnicity-specific sections of the genome may still be missing from our current assemblies. Longer sequencing reads and/or alternate chemistries will become increasingly powerful in perfecting genomic measurements, thereby revealing hidden associations, novel functions, or overlooked DNA modifications.

### Functional mosaicism

DNA sequencing at deep coverage or at single-cell resolution is revealing a vast genetic heterogeneity of normal or dysplastic tissues. At present, these insights are mostly at the stage of observations, but future studies will address the consequences of such heterogeneity in tissue homeostasis and function. The new information that is provided will provide a better understanding of diseases and conditions associated with aging, genotoxic injuries, and the accumulation of such mosaic mutations. Many of these consequences may be dynamic or with limited effect size that become significant over time and will be difficult to study because a longitudinal collection of asymptomatic specimens may lack the relevant phenotypic characterization.

## Jong Bhak, Ulsan National Institute of Science and Technology

Genomics will be everywhere. The history of the universe is divided into two phases: 1) before genome sequencing and 2) after genome sequencing. Understanding our own complete code and being able to manipulate it is the ultimate evolutionary milestone in 14 billion years of evolution. Manipulating or editing whole genomes will bring a completely different view of life. I predict that Darwinian evolution will be heavily modified or even rejected to a certain degree through genomic ‘reading’ and ‘writing’ of life.

In addition, we will have sequencers in our fridges, air conditioners, cars, restaurants, ponds, climate posts, bathrooms, hospitals, and so on. People will see genomics computers or clouds that sense the DNA of the whole Earth in real time at some point. Even today, we already know what kind of flu viruses will be prevalent next year and will know where they actually will be. One of the obstacles presented to genomics is that people fear it or have prejudice against it, just as some fear genetically modified organisms or nuclear reactors. This is not specific to genomics, but general to science and innovation. Science communications will be a key.

## Norbert Perrimon, Harvard Medical School

At present, I am particularly excited by two areas of genomics. The first one is the ability to use CRISPR/Cas9-based methods to perform combinatorial screens effectively in cells. Although the use of combinatorial screening, especially to discover synthetic lethal phenotypes, is not novel, the approach using RNA interference has not been particularly robust and relatively little has emerged from the large investment of many laboratories and companies in this area. CRISPR/Cas9 should help us to realize the promise of combinatorial approaches, which have huge implications for our understanding of the organization of signaling networks and the development of therapeutics. The second exciting area is single-cell RNAseq (scRNAseq) which is revolutionizing our ability to catalog and discover cell types. Soon, we will have reference maps of all of the cells in various organisms, which will allow us to describe cell lineages and mutant phenotypes and also to analyze cellular diversity across organisms.

## Duncan Odom, Cambridge Research Institute

The analysis of genome sequence and the types of functional genomics experiments introduced in the past 10 years are now so widely used as to be routine tools, similar to Western blotting. The many technique variants, also known as ‘XYZ-seq’, are often targeted refinements of previously used methods. As such, they offer comparatively few insights that are truly revolutionary. The next frontier could be novel experimental approaches that quantitatively evaluate the bidirectional connections between genome regulation and the intracellular signaling pathways that re-shape cell function. However, tools that are suitable to interrogate epigenome–signaling interfaces quantitatively are not yet fully developed.

## David Adams, Wellcome Trust Sanger Institute

Imagine knowing how each and every single nucleotide variant (SNV) in the genome influences gene function and cellular phenotypes? This year has seen incredible advances in our understanding of germline-predisposing alleles in genes such as *BRCA1* achieved through the use of saturation mutagenesis [1]. This technology and approaches such as base-editing will see us elucidate the link between variants and gene expression as well as the effect of variants on protein structure, stability, and protein–protein interactions. It should soon be possible to understand how variants alter cellular phenotypes across hundreds of genes. As we scale this technology, we will also be able to explore the role that genetic background and cellular context play in gene function. The surprise of 2018 for me was work performed by my colleagues Iñigo Martincorena and Phil Jones [2], who showed incredible clonal diversity in ‘normal’ non-malignant cells of the esophagus, including numerous driver mutations. This work has many implications, and provokes questions such as what is the genetic make-up of a cancer cell when normal cells may carry many of the important drivers? Why do some people have different landscapes of clones in their esophagus and what does the clonal landscape of other tissues look like? 2019 will be exciting.

## Chrisoph Bock, CeMM Research Center for Molecular Medicine of the Austrian Academy of Sciences

### Functional biology at scale

Research in genome biology is often descriptive in nature—sequencing genomes and meta-genomes, profiling epigenomes and transcriptomes, charting evolutionary history, and cataloging disease-linked risk loci. Thanks to major technological advances, we can now generate such descriptive datasets using high-throughput platforms, but it usually takes a succession of many small-scale experiments to establish true biological function. One grand challenge of genomics research in the next decade is to enable functional biology at scale, finally making the mechanistic dissection of biological processes a high-throughput endeavor and overcoming the ‘one gene, one postdoc’ paradigm of molecular biology. While the latter paradigm has resulted in many fundamental discoveries, it is inherently conservative and leads to strong biases towards a few hundred widely studied genes. If we were able to investigate the functional impact of hundreds or thousands of genes in parallel, we would dramatically reduce our ‘blind spots’ in molecular biology, potentially identifying many new mechanisms and promising drug targets. Forward genetic screening pioneered functional biology at scale several decades ago, and pooled CRISPR screens have recently become one of the most important sources of new biology, despite their very basic readout (guide-RNA counting). Furthermore, CRISPR single-cell sequencing with protocols such as CROP-seq and Perturb-seq has emerged as a new screening paradigm that makes complex transcriptome (or potentially multi-omics) signatures accessible for high-throughput CRISPR screening. These and many other future assays will make it possible to complement the virtues of ‘descriptive genomics’ with high-throughput methods for dissecting biological function.

### The limits of data protection by secrecy

Genomics has emerged as a test case for fundamental questions about data protection and about how societies embrace and regulate new technologies. Genome information is highly identifying and has significant potential for abuse, characteristics that increasingly affect other data types, such as movement profiles recorded by smartphones and vital signs measured by smartwatches. Most notably, genome information is practically impossible to lock away in a safe place—we leave DNA fingerprints wherever we go, and recent technological progress makes it entirely realistic that DNA sequencers will soon become low-cost consumer devices, for example as smartphone add-ons for detecting airborne pathogens or food contamination. Current data protection laws are built on the concept of data sparsity and/or data security, which is effective where data are produced and managed centrally by the government or a small number of large companies. These concepts start to fail for genomics data and will increasingly fail for other data types too. Alternatives to data protection by secrecy have been proposed, including strong anti-discrimination laws and practices as well as hardship funds compensating for damages that individuals incur as the result of data abuse. Such developments are crucial for the population-scale future of genomics research (as pursued by GA4GH and others) that need strong support, acceptance, and embedding in society. Genomics researchers should proactively contribute to the important societal discussions about data protection and personal freedom, sharing their experience with genomics information as one of the most personal, most important, and most difficult to protect types of data.

## Steve Henikoff, Fred Hutchinson Cancer Research Center

Although I admit to having a mediocre record when it comes to crystal-ball gazing, there is little doubt that single-cell genomics will have a major impact on the future of the field. The technology is moving forward rapidly, and at least single-cell RNA-seq is becoming routine enough that it is accessible to developmental biologists, who have much to gain from the transition from population-based to single cell-based genomics. Imaging can provide three-dimensional context to single-cell genomics, and compatible imaging technologies, including super-resolution microscopy, are also advancing rapidly. The major limitations of single-cell genomics are the sparseness per cell and the cost, but experimental and computational approaches continue to chip away at these issues. As single-cell genomic technologies advance, the range of problems that can be addressed using them increases.

## Weida Tong, National Center for Toxicological Research

Toxicogenomics has been struggling to be accepted by the regulatory agencies responsible for the risk and safety assessment of microarray technologies since its inception 18 years ago. The rapid development of next-generation sequencing (NGS) has opened many opportunities beyond microarrays and brought light at the end of tunnel. NGS-based toxicogenomics will address diverse and critical questions that are difficult to address either completely or at all with microarrays. In addition, genomics has contributed significantly to our understanding of the underlying mechanisms of disease and health. I think the future direction will tilt more towards the predictive side by taking advantage of the rapid development of animal-free and high-throughput screening methods (such as the use of in vitro and *induced pluripotent stem cells* (iPSC) methods) in conjunction with artificial intelligence (AI)-based learning.

Two obstacles are worth mentioning.

1. ***Reproducibility***. A challenge that will be continually faced by genomics. Best practice and guidance for genomic data analysis are crucial. We need to develop strategies such as FAIRsharing to reproduce the key figures and results from published studies. We can learn from some journals, such as *Scientific Data* and *GigaScience*, ways to advance the sharing and reuse of scientific data. We also need to encourage new methodologies, such as blockchain, that could help to create incorruptible data trails that will improve reproducibility. ‘Computational reproducibility’ is also an issue as different analysis approaches can give different results and, sometimes, subsequently can lead to very different biology. This phenomenon is bound with the nature of genomics technologies and difficult to avoid. Some baseline practice needs to be recommended.
2. ***Transferability*.** Genomics technologies will continually evolve; today is next-generation and tomorrow will be ‘next next-generation’. I think it is important to think about the question of how today’s gene signature will be sustainable in the future genomics era. There is no general guidance and investigation of this issue is lacking.

## Ling-Ling Chen, Shanghai Institute of Biochemistry and Cell Biology (CAS)

I work on long noncoding RNAs (lncRNAs) with a surprisingly wide range of sizes, shapes, and functions. These features present experimental challenges for their analysis. What isoforms are present and which are functional? How can we precisely map and quantify RNA abundance, isoforms, and different modifications in examined cells, tissues, and developmental stages? How do some lncRNAs accumulate in cis or how are others directed to specific subcellular locations? How do structural motifs and conformations connect to interactions with partners and biological functions? Long-read sequencing will help to decipher individual RNA isoforms as well as post-transcriptional modifications. Genome editing tools will help us to understand the role of individual isoforms, if any. Diverse functions depend on subcellular localization as well as on the formation of structural modules of lncRNAs in partnership with associated proteins, which may undergo rapid changes depending on local or cellular environments. As a consequence, determining the function of RNAs by elucidating the regulation of their expression, their subcellular localization patterns, their interaction partners and the conformation of lncRNAs in time and in space will be critical, and will involve the use of genomics-based approaches in combination with RNA biology.

## Fowzan Alkuraya, King Faisal Specialist Hospital and Research Centre (KFSHRC)/Alfaisal University

How do I see the future of clinical genomics? I think it is safe to bet that whole-genome sequencing for each person will be obtained noninvasively during embryonic development and uploaded to their medical record for a life-long reference. The interaction with this reference will be dynamic based on input from multi-omics. The individual patient will be at the very center of healthcare delivery, and risk calculations will no longer be based on the population average. Somatic genome editing will be routine. Drug development will be primarily driven by our improved understanding of how our genomes influence our health and disease states. Abuse of antimicrobial agents will be all but eliminated by the timely and accurate application of metagenomics. At the same time, individuals will be able to modify their microbiota at will with simple pills and topical agents.

## Detlef Weigel, Max Planck Institute for Developmental Biology

The steep increase in resolution that we have seen in genomics over the past few years will continue, both in terms of individual cells and individual members of a species, and at the temporal and spatial levels. The datasets that result will concern not only transcriptional regulation during development and disease or during environmental fluctuations, but also genomic changes that occur during the evolution of tumors and the evolution of populations. In my specific area of interest, the adaptation of wild plants to the abiotic and biotic environment, I am looking forward to much more fine-grained information about dynamic variation in the size and genetic makeup of a population in response to environmental disturbances, including genetic changes that have occurred in the past, as inferred from the analysis of herbarium specimens. This information will be combined with other data types, such as high-resolution remote imaging, to develop increasingly sophisticated models for forecasting the impact of a rapidly changing environment on many different plant species and ultimately entire ecosystems. It goes without saying that machine-learning methods of all stripes will play a crucial role in building these models.

## Itai Yanai, New York University, and Martin Lercher, Heinrich Heine Universität (HHU) Düsseldorf

The future of genomics may be less about the generation of new data (which will undoubtedly slow) than about how we explore it. Unfortunately, as it is taught, the scientific method relates to hypothesis testing, but tells us nothing about how to generate new hypotheses. Yet some of the largest discoveries in biology—natural selection, the Archaea domain, and neutral evolution, to name a few—emerged from explorations rather than from hypothesis testing. Indeed, exploring complex datasets is one of the most creative endeavors of our time. You are certain that important insights hide beneath the mountain of data in front of you; you just do not know what you are looking for. If you already have a question in mind, AI is amazingly efficient at providing a shortcut to the answer; but now that we have all that data, the most exciting and important part of genomics is to identify the questions that can be answered with it. Enhancing our creativity in genomics will entail building in constraints that frame a particular study, a playful approach to the analysis, brainstorming sessions, and embracing the outliers in the data. Moreover, the most important challenge for creative genomics researchers will be to develop a feel for distinguishing between the big and mundane patterns in the data. We need to nurture such a culture of exploration.

## Shengtao Zhou, West China Second Hospital

Apart from more in-depth genomic investigations in different biological settings, there will be more emphasis on research combining the study of genomics with that of the other omics technologies, including proteomics (not only quantitative proteomics but also post-translational modification (PTM) proteomics), metabolomics, and epigenetic analyses. These multidimensional landscapes of genotypes with an integrated perspective will help us to understand living processes better and will provide insightful guidance for precision medicine in the clinical setting.

## Laurence Hurst, University of Bath

Genomics is in an age of exploration and discovery. Whether we are discovering the genomes of more species, the genomes of more individuals in a species, or more genomes within an individual (at single-cell resolution), we are very much in a phase where we are letting the data lead. This will unquestionably be a rich source of information and will answer many important questions. Some of these questions we do not even know about yet. Indeed, historically, new technologies and new data have been successful at opening up problems that we had not thought about before the data emerged.

Could the future of genomics be different? In evolutionary biology, the tradition has often been the other way around: we start with a problem and a hypothesis and go after the data we need to test the hypothesis. This seems like an enterprise that is being displaced by the drive to sequence. On the broad scale, if there is one thing I would wish for, it would be reaffirmation of hypothesis-driven science, where the drive to sequence (and the decision of what to sequence) is driven beyond a ‘more is better’ or completist approach. Darwin captured this famously in 1861, writing in response to a question about data collection: “About thirty years ago there was much talk that geologists ought only to observe and not theorise; and I well remember someone saying that at this rate a man might as well go into a gravel-pit and count the pebbles and describe the colors. How odd it is that anyone should not see that all observation must be for or against some view if it is to be of any service!” I wonder what Darwin would make of genomics?

For my own part, the questions of interest remain understanding whether genomic activity is mostly so much noise and rubbish or all part of some poorly understood but exquisite machine. This is not just a profound question about the role of selection in evolution, it is also central to predicting which mutations might or might not have a phenotype and potentially cause disease.

## Chuan He, University of Chicago

To map nucleic acid modifications, be they DNA 5mC, 5hmC, or 6 mA, or RNA m6A and other modifications, we really need quantitative methods that not only detect the exact location but also the modification fraction at each modification site in a high-throughput manner. Ideally, the method could be applied to limited input materials and eventually to single-cell studies.

I feel we need to know the proximity information. We want an approach or approaches that effectively map DNA–DNA proximity (Hi-C but more efficient and less costly), RNA–RNA proximity (not much has been done), protein–DNA proximity (ChIP-seq or Cut and Run), protein–RNA proximity (CLIP-seq but more efficient and to the single-cell level), and protein–protein proximity, ideally in a single cell.

Genomics will be applied in the future to clinical disease diagnosis and prognosis. Liquid biopsy for early disease detection will be a huge advance in healthcare and I believe that this will be feasible in the next few years.

Many tools in genomics were developed for basic research. They may not be ready for direct applications in clinical practice. For example, the most common RNA-seq approach would demand RNase-free operations, which would be difficult to achieve in clinics. We have yet to focus on developing clinically friendly genomics tools, leaving plenty of space for innovation.

## Claudia Köhler, Swedish University of Agricultural Sciences

I think the major challenge in my area of research is to sequence and assemble centromeric regions. These regions are still largely unknown and, because of their highly repetitive nature, are difficult to sequence and assemble. However, long-read sequencing, such as the sequencing offered by Nanopore, is close to overcoming this obstacle and I can imagine that the resulting data will offer exciting new insights into the functional role of centromeres. Centromeres are fast evolving and play a major role in speciation, so understanding how they are composed and what drives their formation is of major biological importance.

## Lluis Quintana-Murci, Institut Pasteur

We have exciting years in genomics and population genetics in front of us, particularly in the era of whole-genome sequencing at the population scale, and now that we are able to obtain population data from ancient genomes from different times and geographic transects. These datasets, together with a needed improvement in methods to detect selection, will enable us to understand in further depth the way that humans can adapt to environmental changes in the long run (through genetic changes) or in the short run (through epigenetic changes?...and this is a real ‘question mark’). We thus need to develop more empowered approaches to detect different types of selection, such as polygenic adaptation or adaptation through admixture (both with archaic humans but also, in particular, with other modern human populations). All of these questions in population genetics will continue to help us to pinpoint regions of the genome that have contributed to human survival over time, and that should be involved in phenotypic diversity, either benign or disease related. Another exciting area of population genetics is the possible tradeoffs of some past events of positive selection, which are today associated with maladaptation.

## Chris Mason, Weill Cornell Medicine

**Fig. 1:**
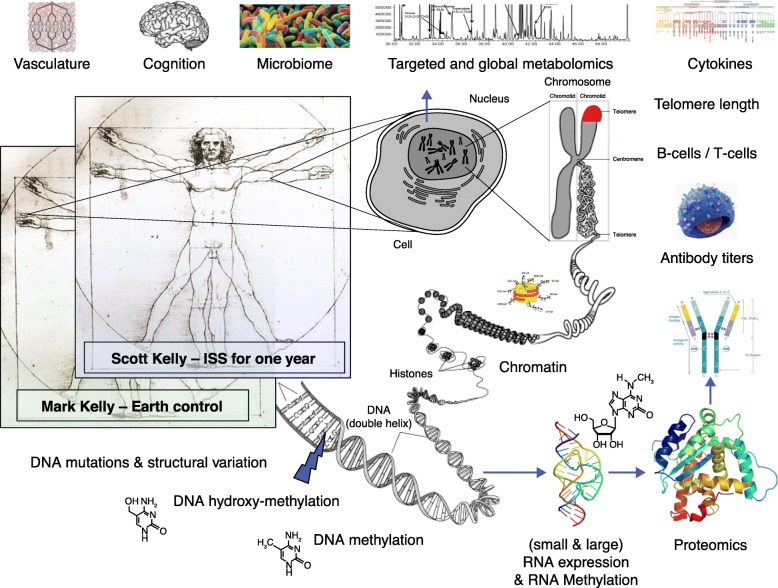
Multi-omic measures from the NASA Twins study. Work from the NASA Twins Investigators Group established a first draft multi-omics profiling and data integration framework that included data from the genome (DNA), epigenome (modified DNA and chromatin states), transcriptome (RNA), epitranscriptome (modified RNAs), proteome (LC-MS), metabolome (GC-MS), antibody profiling and VDJ recombination, B-cell/T-cell counts and sequencing, telomere length (FISH, ddPCR), cytokine measures, targeted and global metabolomics, microbiome and metagenome, cognition, and vasculature dynamics

The continued eruption of sequencing data across all cell types, modalities of biology, and branches of phylogeny is approaching Yottabyte-scale. This has led to the birth of entirely new disciplines such as the epitranscriptomics [3], to the integrated interrogation of multi-omic metrics (Fig. 1), and even to new technologies for rapid sequencing in zero gravity [4]. These developments enable a temporal and kingdom-agnostic view of genomics, whereby we can learn from species that died long ago (through ancient DNA) to help those today (conservation). These methods and tools help genome mapping projects (such as the Global Alliance for Genomics and Health (GA4GH)), genome assembly projects (such as the Vertebrate Genomes Project (VGP)), and clinical genomics projects (such as ClinVar). Spatiotemporal maps, including those produced by the Earth Microbiome Project, urban DNA in MetaSUB, the Earth BioGenome Project, and the Tara Oceans Project, also span far beyond eukaryotes. Together, these data are quite literally creating a genetic catalog of our planet’s present and past.

But, what is even more exciting than all the data and the discoveries of today is what comes next: the first-ever inter-planetary genomics experiment. In 2020, the Mars2020 mission will preserve Martian rocks on the surface of Mars for return to NASA. When the samples return to Earth in the late 2020s, some of these rocks will be sequenced. The first data that come from any possible nucleic acid in those samples will be cross-referenced with any fragment of DNA or RNA ever observed on Earth, creating a planetary-scale genomic filter, and they will also be compared to the genetic catalog of contaminants being aggregated in the spacecraft assembly rooms at the Jet Propulsion Laboratories (JPL). Thus, in the near future, we will witness the birth of ‘Epoch Filters’ for ancient DNA and ‘Planetary filters’ for k-mers and observed in sequences from one planet or another. Then, with missions that will send probes to other planets’ moons, like the Europa Clipper, it will be possible to sketch a genetic map of planets and a system, which will be needed to scan for any signs of life before we get there.

## Michael Schatz, Johns Hopkins University

On 14 April 2003, after more than 10 years of work, and billions of research dollars spent, the human genome project was declared completed. With it, the complete set of genes and other genetic information for our species was known for the first time. At the announcement ceremony at the conclusion of the project, President Bill Clinton remarked ‘Without a doubt, this is the most important, most wondrous map ever produced by humankind.’ In the years since, countless studies have benefited from this tremendous resource while exploring aspects of human evolution, human biology, and disease.

As important and wondrous this map has been, it suffers from two major shortcomings. First, the human reference genome is not actually complete, and over one hundred and fifty million nucleotides still remain undetermined and other regions are incorrectly represented [5]. Second, the reference human genome does not actually represent any specific human, and is instead a mosaic of many individuals [6]. This can distort the interpretation of individual genome sequences as we all carry millions of differences from the reference.

Fortunately, new single-molecule DNA sequencing and mapping biotechnologies [7] are beginning to make it possible for us to move away from a single reference genome and towards personalized genomes for everyone. To achieve this, we need improved sequencing and base-calling methods to convert raw electrical or optical signals into a faithful representation of the sequences. Random errors are relatively straightforward to overcome, but any systematic errors will remain. Next, we need more efficient and more accurate de novo and reference-guided assembly methods to build the personalized genome sequences over massive scales. Great gains have been made in the past few years, but more work is needed, especially to resolve repetitive and heterozygous sequences fully.

I am particularly excited to use these technologies to study the role of structural variations in health and disease, something that remains almost entirely invisible to standard short-read sequencing [8]. With these obstacles overcome, the next phase of genomics will focus largely on the interpretation of personalized genome sequences. Two of our best tools for this are comparative genomics, which allows us to see which individuals share the same variants, and functional genomics, which measures how those variants impact gene expression and regulation. Both would benefit from the development of additional approaches for pan-genome analysis, especially graph genome technologies that can represent large collections of personalized genomes in a single integrated data structure [9, 10]. I predict that with these approaches we will come to realize the rules that nature has created for expressing phenotypes out of genotypes is more wondrous and more organized than is currently recognized.

At *Genome Biology*, we are looking forward to seeing all of this happen, and more!
